# Evolving spiking neural networks: the role of neuron models and encoding schemes in neuromorphic learning

**DOI:** 10.3389/fnins.2026.1697163

**Published:** 2026-02-06

**Authors:** Bastian Loyola-Jara, Gabriela Fernández-Rodríguez, Javier Baladron

**Affiliations:** Departamento de Ingeniería Informática, Universidad de Santiago de Chile, Santiago, Chile

**Keywords:** artificial intelligence, evolutionary computation, neuromorphic engineering, reinforcement learning, spiking neural networks

## Abstract

This study investigates the impact of neuron models and encoding schemes on the performance of spiking neural networks trained using the NeuroEvolution of Augmenting Topologies (NEAT) algorithm. By evaluating both classification and reinforcement learning tasks, we compare the performance of the Leaky Integrate-and-Fire (LIF) and Izhikevich neuron models across various input and output coding strategies. Our results demonstrate that the Izhikevich model consistently outperforms the simpler LIF model, except in one task where both showed comparable results. These findings emphasize that the choice of neuron model is as critical as encoding schemes in neuromorphic learning and highlight the importance of task-specific configuration. The study also showcases the potential of simulation frameworks for prototyping and optimizing neuromorphic systems.

## 1 Introduction

At the core of neuromorphic computing lies the architecture of spiking neural networks (SNNs), which emulate the temporal dynamics and event-driven communication observed in biological neural systems. These networks offer a promising framework for energy-efficient, real-time processing, especially in edge and embedded systems. However, training SNNs remains a significant challenge. Neural network training is inherently a difficult optimization problem, typically addressed through gradient-based methods such as backpropagation (Kaveh and Mesgari, 2023; Froese and Hertrich, 2023). In SNNs, the activation functions of spiking neurons are non-differentiable due to their discontinuous firing behavior. To overcome this, several surrogate gradient methods have been developed to approximate gradients during training (Neftci et al., 2019). While effective in enabling backpropagation-like optimization, these methods lack biological plausibility and may limit the flexibility and generalization capabilities inherent to brain-like computation, particularly in transfer learning and multitask scenarios.

Evolutionary algorithms present an alternative, gradient-free paradigm for training SNNs (Schuman C. D. et al., 2022). These approaches optimize network parameters, such as synaptic weights and connection topology, through population-based search and stochastic variation (Abd Elaziz et al., 2021). Evolutionary strategies have a long-standing history in machine learning and artificial intelligence, where they have been successfully applied to evolving deep neural networks without the need for gradient information (Mishra and Kane, 2023; Galván and Mooney, 2021).

In the context of SNNs trained with evolutionary methods, recent research highlights the crucial role of spike encoding and decoding schemes in determining network performance (Auge et al., 2021). Two primary approaches have emerged: rate-based and temporal-based encoding. For example, Schuman et al. (2019); Schuman C. et al. (2022) systematically explored multiple encoding and decoding strategies across classification, regression, and control tasks. Their findings demonstrate that the optimal encoding strategy is task-dependent. Notably, their evaluations were based on the simple leaky integrate-and-fire (LIF) neuron model, which has been widely adopted in early neuromorphic hardware due to its computational simplicity and hardware efficiency.

However, modern neuromorphic platforms are increasingly capable of supporting more biologically realistic neuron models, such as the Izhikevich model. This model combines computational efficiency with the ability to replicate a wide range of biologically observed spiking behaviors using only a few parameters. For example, the ODIN chip (Frenkel et al., 2018) is an open-source digital neuromorphic processor capable of emulating the 20 distinct biological firing patterns of the Izhikevich neuron. Beyond digital systems, photonic implementations (Lee et al., 2022) and resistive random-access memory (RRAM)-based platforms (Jiang et al., 2023) have been proposed to implement Izhikevich-type dynamics in energy-efficient, high-speed hardware. More recently, the Darwin3 chip (Ma et al., 2024) has been introduced as a highly scalable neuromorphic system. While not yet fully evaluated with the Izhikevich model, Darwin3 is designed to support arbitrary two-dimensional neuronal dynamics, offering future compatibility with complex neuron models. The adaptability of the Intel Loihi chip has been shown to be strong enough to run the Izhikevich model efficiently (Uludağ et al., 2024).

In this study, we investigate the impact of neuron models on the performance of SNNs trained using an evolutionary algorithm that jointly optimizes network structure and synaptic weights. Our experiments span both classification and reinforcement learning problems, allowing us to explore how the interplay between encoding/decoding strategies and neuron model dynamics affects learning outcomes. The results demonstrate that, in addition to choosing appropriate encoding schemes, the neuron model itself plays a critical role in determining the network performance. These findings emphasize the need for hardware-software co-design in neuromorphic systems and motivate the development of neuromorphic hardware capable of efficiently simulating a broader range of complex spiking neuron models.

## 2 Materials and methods

For all experiments, the NeuroEvolution of Augmenting Topologies (NEAT) algorithm was used to simultaneously evolve the weights and structure of spiking neural networks (Papavasileiou et al., 2021; Stanley and Miikkulainen, 2002). The algorithm operates on a population of candidate solutions, in this case, spiking neural networks, designed to solve a specific task. In each generation, the networks are first evaluated based on their performance on the target problem. A selection process then removes the least fit individuals, and the remaining population is modified using stochastic genetic operators. Mutation operations include altering synaptic weights, adding new nodes, or inserting new connections. Crossover operations recombine the structure and parameters of two parent networks to generate offspring. The probability of applying each operator is controlled by predefined parameters, enabling a balance between exploration and exploitation throughout the evolutionary process.

NEAT has already been demonstrated as an effective method for training spiking neural networks. For example, Qiu et al. (2018) used NEAT to evolve SNNs capable of solving nonlinear control problems in continuous domains. Elbrecht and Schuman (2020) employed a variant of NEAT to train SNNs on a range of reinforcement learning and classification benchmarks. Similarly, Custode et al. (2022b) evolved networks that successfully solved classic control tasks from the OpenAI Gym suite. In a related study, Custode et al. (2022a) applied NEAT to train SNNs for predicting the remaining useful life of mechanical systems.

We extended the ANNarchy neural simulator framework (Vitay et al., 2015) by implementing the NEAT algorithm to evolve spiking neural networks. Our implementation manages a population of candidate networks, evaluating each within the ANNarchy environment at every iteration. During the evaluation phase, candidate networks are distributed across available processors for parallel execution, optimizing computational efficiency. The implementation is publicly accessible at our repository including a set of interactive notebooks illustrating its use.

The spiking neural networks evolved in this study do not follow a predefined layered architecture. Instead, network topology is entirely determined by the NEAT algorithm, which evolves both the presence and strength of synaptic connections. Networks are initialized with a minimal structure and progressively augmented through mutation operators that add neurons and synapses during evolution.

From an architectural perspective, the resulting networks can be described as recurrent spiking networks with potentially all-to-all connectivity, where the final connectivity pattern is selected by evolutionary optimization. No explicit separation between input, hidden, and output layers is enforced beyond the functional role of the corresponding neuron populations. This design allows the emergence of task-specific recurrent dynamics rather than imposing a fixed feedforward or reservoir-based structure.

All synaptic projections are implemented using a single projection type within the ANNarchy framework. While projections are defined as excitatory at the model level, synaptic weights are real-valued and can take both positive and negative values as determined by the evolutionary process. Consequently, the evolved networks may exhibit both excitatory and inhibitory effective interactions, despite the absence of explicitly distinct inhibitory neuron populations.

Although this architectural flexibility shares certain similarities with reservoir computing approaches such as Liquid State Machines, the networks studied here do not satisfy the defining constraints of LSMs, such as fixed random connectivity or separation between a static reservoir and a trained readout. Instead, both connectivity and dynamics are shaped by evolution, resulting in task-adaptive recurrent networks rather than a predefined computational substrate.

We adopt the original speciation mechanism from NEAT, where genomes are grouped into species based on the following compatibility distance:


$$\begin{array}{l} \delta =\frac{c1E}{N}+\frac{c2D}{N}+c3\bar{W} \end{array}$$ (1)

where, *E* and *D* denote the number of excess and disjoint genes, respectively, i.e., non-matching genes that lie outside or within the range of the other parent's innovation numbers. *N* is a normalization factor, and $\bar{W}$ represents the average weight difference of matching genes. A genome is assigned to a species if its distance to the representative genome of that species is below a predefined threshold. Priority is given to crossovers between solutions of the same specie.

We conducted a series of experiments to generate neuromorphic agents capable of solving classic reinforcement learning benchmarks available through the Farama Gymnasium API. For each task, we evaluated the fitness of each candidate solution by running 100 episodes and calculating the mean return. Agents producing higher cumulative rewards were thus favored by the algorithm. In each step of an episode, the current state was represented as spiking activity in the input neurons using two distinct encoding schemes. The first scheme employed two neurons per state feature: one representing positive values and the other representing negative values. The input current of the relevant neuron was modulated based on the feature value. This scheme also used two output neurons, each corresponding to a possible action. The action with the highest number of spikes within 50ms of simulation was selected.

The second encoding scheme was based on that of Haşegan et al. (2022) and used 20 neurons per state feature, with each neuron corresponding to a specific interval of the feature's possible values. The center of each interval was samples from a Gaussian distribution. In the first set of experiments, if the current feature value fell within the range assigned to a neuron, that neuron emitted a single spike. In the second set, the input current to each neuron was increased to 75 for LIF neuron model and 20 Izhikevich model, inducing the neuron to generate multiple spikes. This scheme considered 20 output neurons, 10 for each action. The action with more spikes after 50ms of simulation was selected.

All encoding schemes were evaluated using both the Leaky Integrate-and-Fire (LIF) and the Izhikevich neuron models for each task. In the LIF model, the membrane potential *v* evolves according to a first-order differential equation, in which incoming spikes are integrated and the potential decays exponentially over time:


$$\begin{array}{l} \tau \frac{dv}{dt}=-v+g+I \end{array}$$ (2)

The Izhikevich model, by contrast, is a more complex and biologically plausible system defined by a pair of coupled differential equations:


$$\begin{array}{ll} \frac{dv}{dt} & =0.04v+5v+140-u+I+g \end{array}$$ (3)

$$\begin{array}{ll} \frac{du}{dt} & =a\left(bv-u\right) \end{array}$$ (4)

Here, *v* represents the membrane potential and *u* is a recovery variable that accounts for membrane recovery and after-spike behavior. This model is capable of reproducing a wide range of neuronal firing patterns observed in electrophysiological recordings by tuning its parameters.

In both models, when the membrane potential *v* reaches a predefined threshold, a spike is emitted and propagated to all connected neurons. Upon receiving a spike, the synaptic conductance *g* is increased by a fixed synaptic weight. Between spikes, *g* decays exponentially toward zero. After a spike the membrane potential is reset to –65. Additionally, the recovery variable u is increased by a fixed amount (d = 8) after each spike, following the standard regular-spiking neuron parameterization originally proposed by Izhikevich (2003). This increment controls the strength of spike-frequency adaptation and was kept constant across all experiments to ensure a fair comparison between neuron models.

For each combination of encoding scheme and neuron model, we optimized the NEAT hyperparameters using Bayesian optimization via the Optuna framework (Akiba et al., 2019). Specifically, we employed the Tree-structured Parzen Estimator algorithm within Optuna to sample 100 hyperparameter combinations sequentially. The considered hyperparameters are shown in Table 1. For all parameters, a network is considered large if it has more than 20 neurons. We selected the hyperparameters that produced the highest-performing network for each encoding scheme and evaluated it over 31 additional runs with a population of 50 networks and ran for 50 generations.

**Table 1:** Hyperparameters considered in the optimization done for each model and coding configuration.

**Name**	**Description**	**Range**
K	Percentage of solutions that are kept for the next generation	40%–60%
T	Compatibility threshold to include a solution into a specie	2.0–4.0
N	Probability that a new genome is created though a mutation	0.15–0.35
*P*_*w*_	Probability of changing a weight in a network	0.7–0.9
$P_{n}^{s}$	Probability of adding a neuron in a small network	0.02–0.04
$P_{c}^{s}$	Probability of adding a connection in a small network	0.01–0.05
$P_{n}^{l}$	Probability of adding a neuron in a large network	0.02–0.04
$P_{c}^{l}$	Probability of adding a connection in a small network	0.01–0.05
*C*1	C1 parameter of the distance measure	0.5–1.5
*C*2	C2 parameter of the distance measure	0.5–1.5
*C*3	C3 parameter of the distance measure	0.3–0.5

We conducted a second set of experiments to develop neuromorphic classifiers capable of solving benchmark problems from the UCI Machine Learning Repository. For this purpose, we adapted the input and output coding strategies identified as effective by Schuman C. et al. (2022) for classification problems.

In the first input coding scheme, the range of each feature was divided into five equal intervals, with each interval assigned to a distinct input neuron. A neuron emitted a single spike at the beginning of the simulation if the corresponding feature value fell within its designated range. In the second scheme, a temporal encoding strategy was employed. In this temporal encoding scheme, the value of each feature was mapped to a spike latency, with higher values producing longer delays before spike emission. This mapping was chosen to maintain a monotonic relationship between feature magnitude and spike time, rather than to strictly mimic biological latency coding.

Two output encoding schemes were implemented also based on those identified by Schuman C. et al. (2022). In the voting mechanism, one output neuron was assigned to each class, and the predicted class corresponded to the neuron that emitted the most spikes. In the first-spike mechanism, the predicted class was determined by the first output neuron to emit a spike.

For each classification problem, the corresponding dataset was loaded and normalized so that all feature values fell within the range [0, 1]. During evaluation, 50 subsets were generated by randomly sampling 50 data points from the original dataset (with replacement), allowing individual samples to appear in multiple subsets. Each candidate network was evaluated on all subsets, and its average performance was used as its fitness score.

As in the reinforcement learning experiments, we performed Bayesian optimization using the Optuna framework to tune the NEAT hyperparameters for each combination of input and output encoding strategy and neuron model. A total of 100 parameter sets were evaluated, and the best-performing configuration was then used in 31 independent runs to assess performance stability and generalization. Each independent run considered a fixed population size of 50 networks and 50 generations.

## 3 Results

### 3.1 The Cartpole problem

The Cartpole problem is a classic benchmark task in reinforcement learning and control systems. The goal is to balance a pole upright on a moving cart by applying forces to the cart, typically to the left or right. This problem is widely used to test and demonstrate reinforcement learning algorithms because it requires learning a policy to control a dynamic, unstable system.

**Figure 1:**
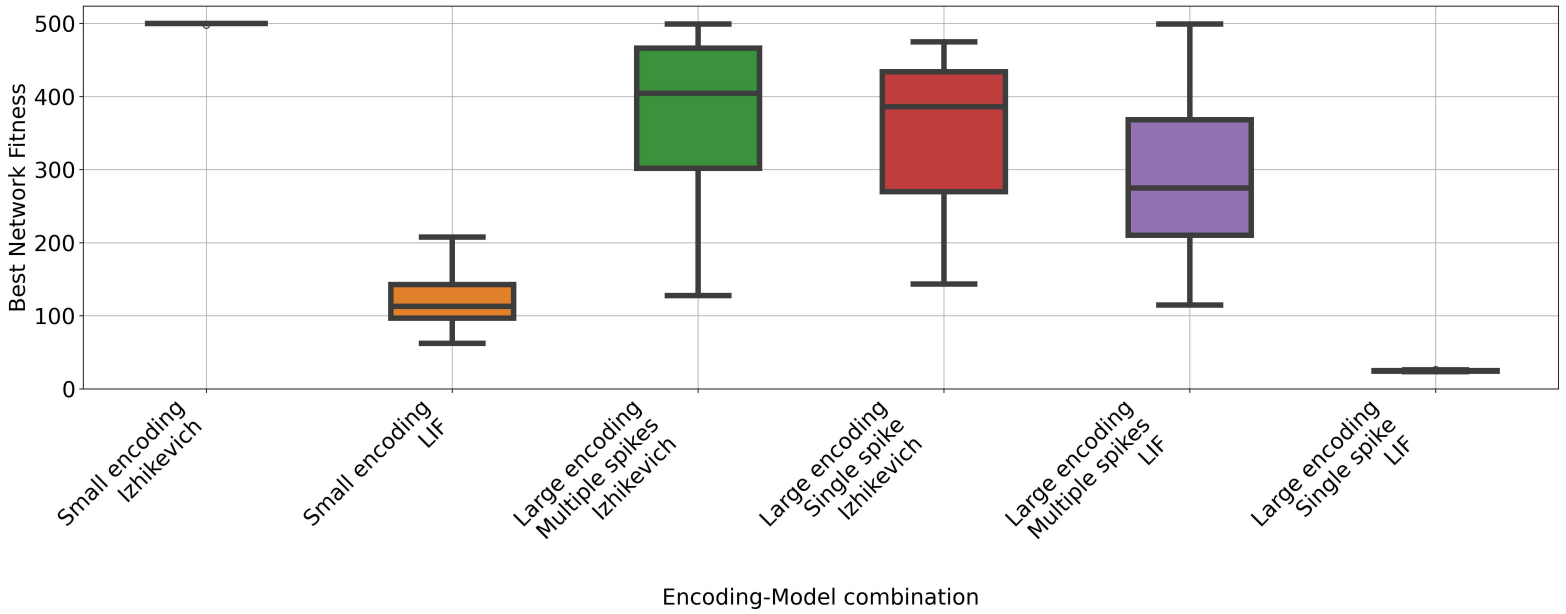
Rewards obtained by neuromorphic agents using different combinations of coding strategies and neuron models on the CartPole task. Each box represents the results of 31 independent runs using the best parameters found through a Bayesian Optimization process. The maximum possible reward is 500.

A fitness function was created using the Cartpole problem implementation available in Gymnasium. Then, a comparison was conducted across all combinations of encoding schemes and neuron types (see Figure 1 and Table 2). The best performance was observed when using the Izhikevich neuron model paired with an encoding scheme that assigned two neurons per feature of the state space. With this configuration, the NEAT algorithm consistently generated networks capable of achieving the maximum possible reward (500). In contrast, substituting the neuron type while maintaining the same encoding scheme resulted in a reduction in the rewards attained by the networks. A Mann-Whitney U test performed between all pairs of groups sharing the same encoding scheme but differing in neuron model revealed significant differences across all comparisons.

**Table 2:** Mean return obtained in the Cartpole problem for each configuration.

**Encoding**	**Model**	**Mean**	**STD**
Small encoding	Izhikevich	499.93	0.35
Small encoding	LIF	120.45	34.76
Large encoding Multiple spikes	Izhikevich	375.95	103.08
Large encoding Single spike	Izhikevich	343.76	106.22
Large encoding Multiple spikes	LIF	289.13	105.54
Large encoding Single spike	LIF	24.69	0.66

Results are obtained from 31 independent runs with the best hyperparameter found for each configuration.

**Figure 2:**
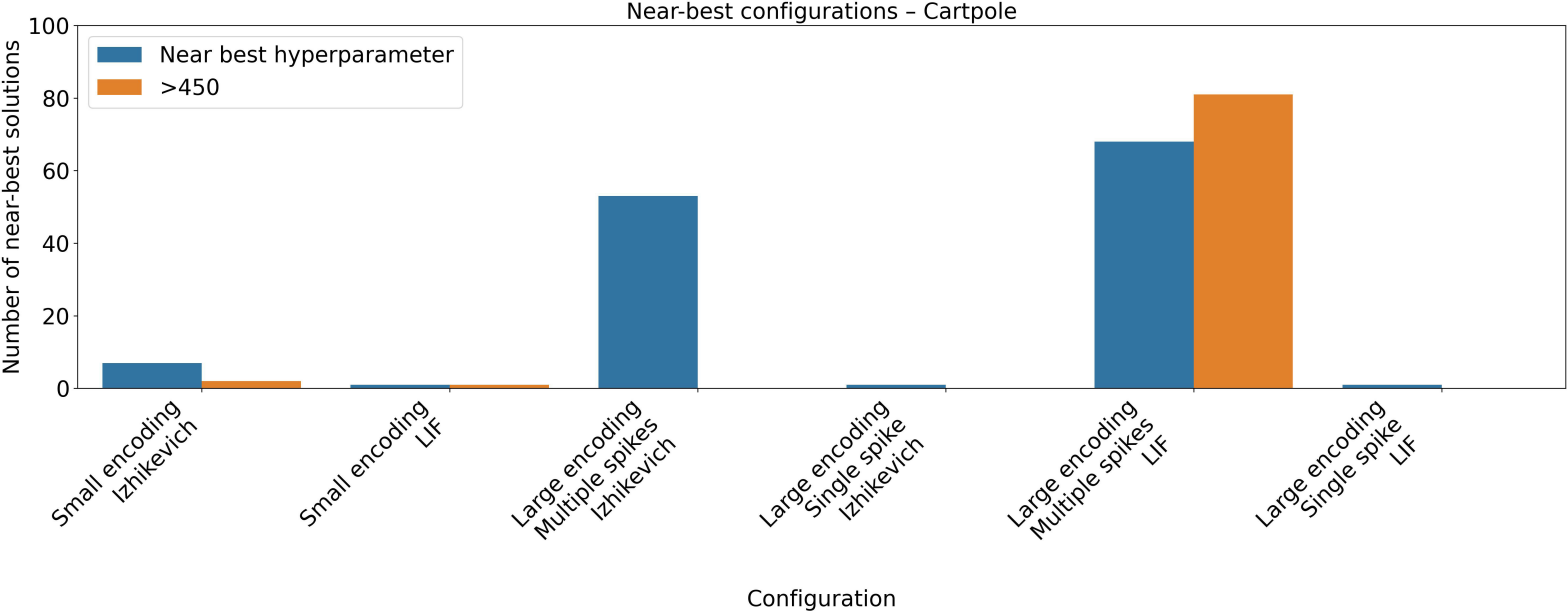
Near-best analysis for the CartPole task. Hyperparameter optimization was carried out using the Optuna framework, evaluating 100 hyperparameter configurations for each combination of neuron model and encoding scheme. The figure reports the number of configurations achieving a mean return within 95% of the best reward obtained during optimization, as well as those whose performance exceeded 450. The maximum achievable reward in the task is 500.

The hyperparameter search for the cartpole task showed only a small number of configuration with high performance (see Figure 2 and Table 3) for all configurations except on those where the large encoding scheme with multiple spike was used.

**Table 3:** Hyperparameter values found for the Cartpole Problem.

**Config**.	**K**	**T**	**N**	***P*_*w*_**	** $P_{n}^{s}$ **	** $P_{c}^{s}$ **	** $P_{n}^{l}$ **	** $P_{c}^{l}$ **	***C*1**	***C*2**	***C*3**
Small-IZH	0.577	3.241	0.329	0.851	0.0305	0.0436	0.0417	0.0789	0.53	0.959	0.306
Small-LIF	0.463	3.79	0.295	0.847	0.0336	0.015	0.0733	0.16	0.981	0.94	0.41
Large-Mult.IZH	0.55	3.296	0.23	0.732	0.039	0.012	0.37	0.125	1.237	1.215	0.418
Large-Sin.IZH	0.48	3.722	0.27	0.813	0.027	0.025	0.177	0.115	0.977	0.743	0.378
Large-Mult.LIF	0.463	2.195	0.199	0.781	0.037	0.026	0.369	0.122	1.362	1.498	0.386
Large-Sin.LIF	0.443	2.083	0.299	0.895	0.033	0.044	0.168	0.173	1.436	1.057	0.426

### 3.2 The Acrobot problem

The Acrobot problem is another well known classic control benchmark task used in reinforcement learning. In this problem an agent must control a system composed of two linearly connected links forming a chain, with one end securely fixed. The goal is to swing chain above a given height. We followed the same method as in the previous task, implementing a fitness function using Gymnasium, optimizing hyperparameters for each possible configurations and then comparing them.

**Figure 3:**
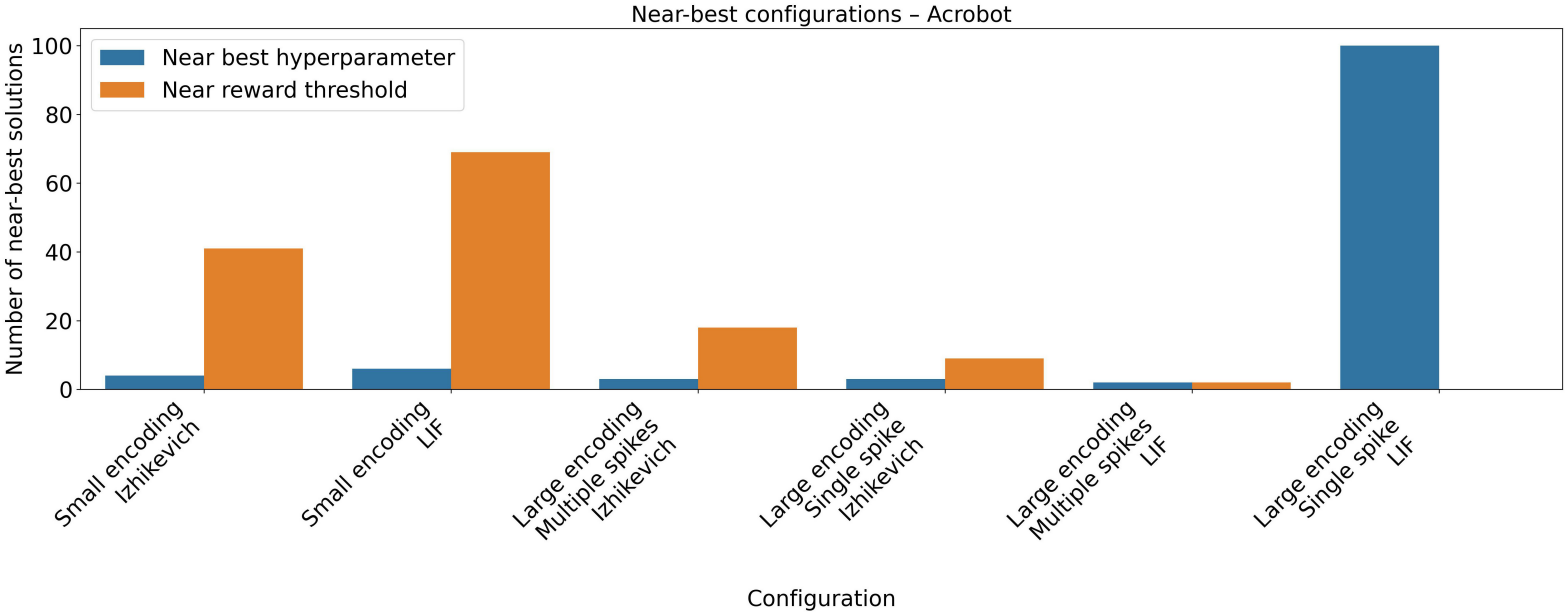
Near-best analysis for the Acrobot task. Hyperparameter optimization was performed using the Optuna framework, evaluating 100 different hyperparameter configurations for each combination of neuron model and encoding scheme. The plot shows the number of configurations that achieved a mean return within 95% of the best reward obtained during the optimization, as well as the number of configurations whose performance exceeded 95, corresponding to 95% of the 100 reward threshold defined in the Gymnasium environment. The best hyperparameter configuration identified during the search achieved a mean return above the task's reward threshold, indicating successful task resolution.

The hyperparameters found for the Acrobot task can be seen in Table 4. A near-best analysis indicates that the hyperparameter optimization identified multiple high-performing configurations when the small encoding scheme was used with both the LIF and Izhikevich neuron models (see Figure 3). In contrast, for the large encoding scheme, only a limited number of hyperparameter configurations achieved comparable performance, suggesting a narrower region of optimal solutions in the hyperparameter space.

**Table 4:** Hyperparameter values found for the Acrobot Problem.

**Config**.	**K**	**T**	**N**	***P*_*w*_**	** $P_{n}^{s}$ **	** $P_{c}^{s}$ **	** $P_{n}^{l}$ **	** $P_{c}^{l}$ **	***C*1**	***C*2**	***C*3**
Small-IZH	0.456608	3.93036	0.301752	0.797799	0.0356973	0.027703	0.384039	0.0849447	1.44458	1.37687	0.439188
Small-LIF	0.518047	3.83971	0.285955	0.854883	0.027212	0.0320672	0.0564179	0.12208	0.866758	0.85722	0.382126
Large-Mult.IZH	0.444614	2.41615	0.287127	0.865444	0.0328525	0.03676	0.165754	0.155157	1.16114	0.633664	0.329576
Large-Sin.IZH	0.516751	3.86131	0.18024	0.75268	0.0224627	0.0256882	0.209747	0.142495	1.3639	0.527036	0.331273
Large-Mult.LIF	0.439851	3.79455	0.232286	0.793278	0.030175	0.043842	0.104855	0.133227	0.811276	1.41175	0.400393
Large-Sin.LIF	0.41281	3.80242	0.315768	0.764831	0.0382941	0.0261577	0.309795	0.179674	0.516195	1.43995	0.484419

**Figure 4:**
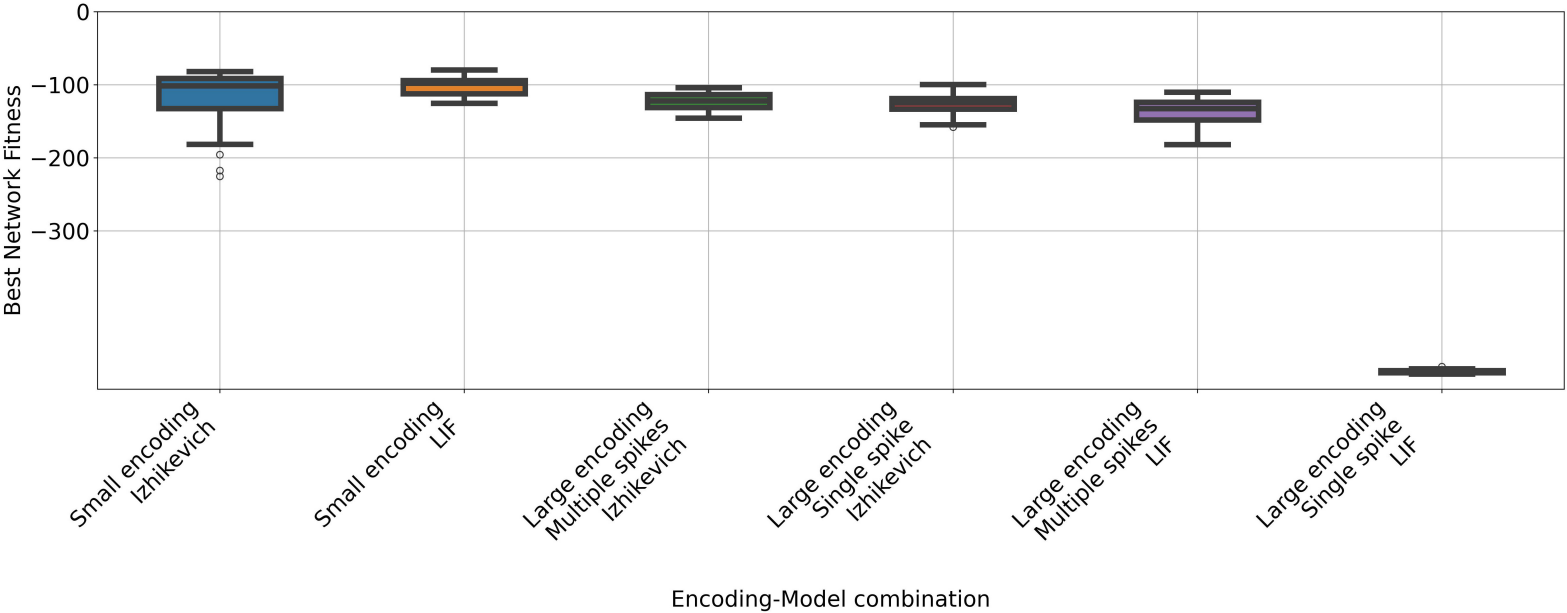
Rewards obtained by neuromorphic agents using different combinations of coding strategies and neuron models on the Acrobot task. Each box represents the results of 31 independent runs using the best parameters found through a Bayesian Optimization process. The task being is considered solved if a reward of –100 is achieved.

The performance differences between configurations were much smaller in this task (see Figure 4 and Table 5). The evolutionary algorithm successfully generated networks that solved the task using both the LIF and Izhikevich neuron models with the simpler encoding strategy. However, the LIF-based models exhibited lower variability in performance compared to those using the Izhikevich model. With the encoding configuration that considers 20 neurons per feature and only one spike per neuron, the algorithm was unable to find proper networks.

**Table 5:** Mean return obtained in the Acrobot problem for each configuration.

**Encoding**	**Model**	**Mean**	**STD**
Small encoding	Izhikevich	–120.24	41.10
Small encoding	LIF	–101.32	12.21
Large encoding Multiple spikes	Izhikevich	–122.34	11.84
Large encoding Single spike	Izhikevich	–126.72	12.96
Large encoding Multiple spikes	LIF	–136.52	18.03
Large encoding Single spike	LIF	–492.62	2.31

Results are obtained from 31 independent runs with the best hyperparameter found for each configuration.

A Mann-Whitney U test on the data of the executions that used the simpler encoding revealed no significant difference between networks employing the Izhikevich and LIF models. In contrast, a significant difference was observed between the two groups using the larger, multi-spike encoding, indicating that under this configuration, networks with Izhikevich neurons outperform those with LIF neurons.

### 3.3 The Mountain car problem

In the Mountain Car problem, the agent controls a car initially positioned in a valley between two hills. The objective is to drive the car to the top of the right hill. Since the car's engine is not powerful enough to climb the hill directly, the agent must learn to build momentum by moving back and forth, choosing when to accelerate left or right.

**Figure 5:**
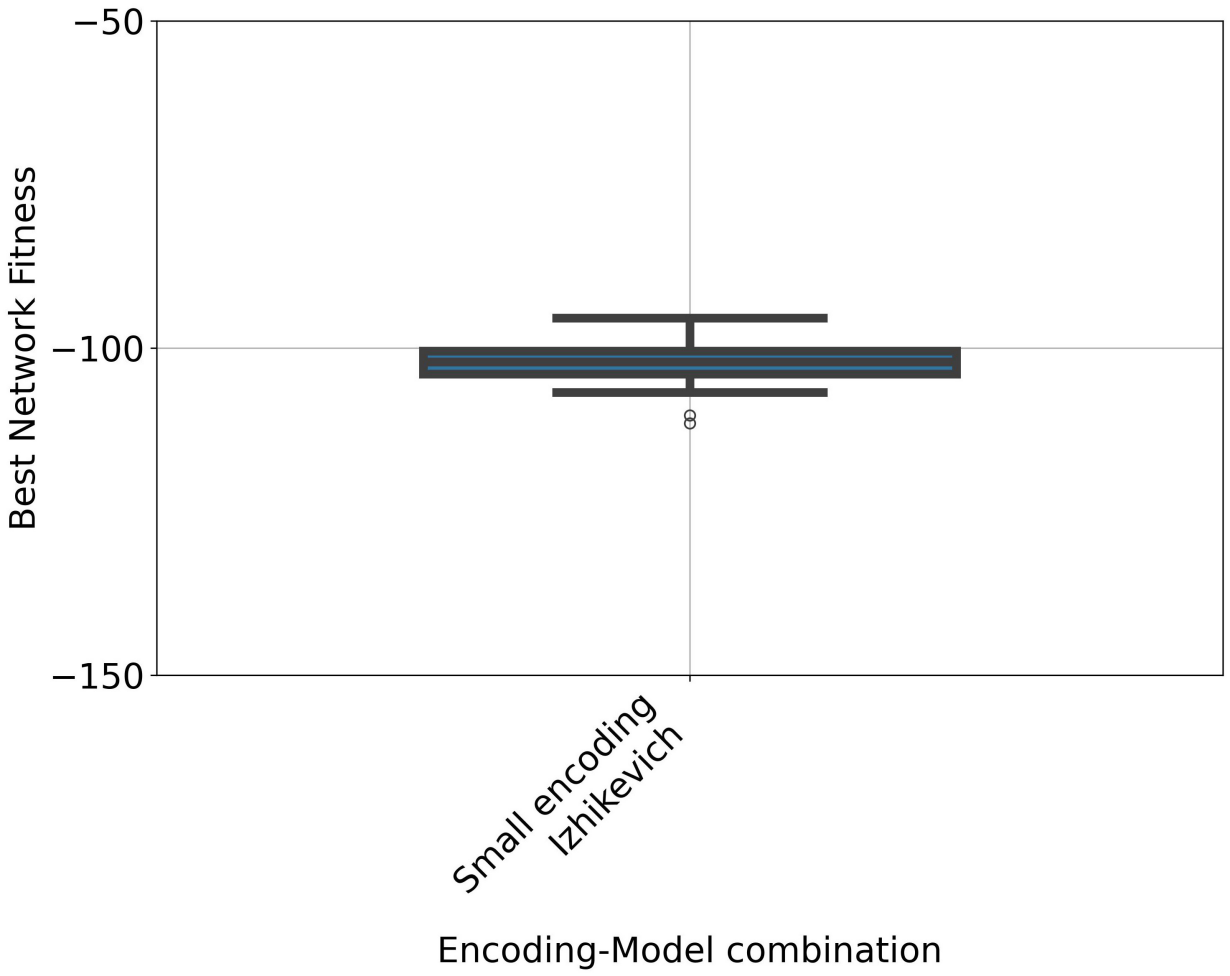
Rewards achieved by neuromorphic agents employing the Izhikevich neuron model and the compact encoding scheme on the Mountain Car task. The boxplot summarizes results from 31 independent runs using the optimal parameters identified via Bayesian Optimization. The task is considered solved when the agent attains a reward of –100 or higher.

We applied the same procedure to the Mountain Car task. Among all tested configurations, only the combination of the small encoding scheme with the Izhikevich neuron model yielded networks capable of solving the task (see Figure 5), achieving a mean return of −102.25 with a standard deviation of 3.57. For all other configurations, the hyperparameter search consistently yielded a return of −200, which corresponds to the minimum achievable reward in the Mountain Car task. This outcome indicates that the evolved agents failed to escape the initial valley and remained close to their starting position throughout the episode.

### 3.4 The Iris dataset

**Figure 6:**
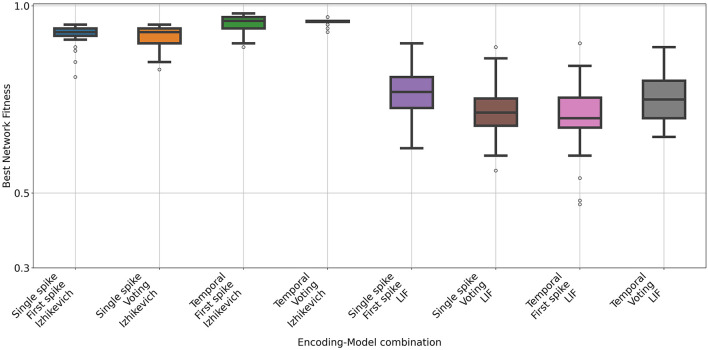
Accuracy of the spiking networks obtained for the Iris dataset. Each box shows the results of 31 independent runs with different input, out coding and neuron model. Configurations with the Izhikevich neuron model consistently performed better than those with LIF.

The Iris dataset contains 50 instances of different types of iris plants. The spiking networks generated for the Iris dataset with the Izhikevich model achieved accuracy values exceeding 0.92 (see Figure 6, Tables 6, 7 for hyperparameter values). These results are comparable to the state-of-the-art performance for each respective problem. The networks obtained with the LIF model achieved lower accuracy values as confirmed with a Mann-Whitney U test between all pairs of data with same input and output configuration.

**Table 6:** Mean accuracy obtained in the Iris dataset for each configuration.

**Input coding**	**Output coding**	**Model**	**Mean**	**STD**
Single spike	First spike	Izhikevich	0.92	0.03
Single spike	Voting	Izhikevich	0.92	0.02
Temporal	First spike	Izhikevich	0.94	0.02
Temporal	Voting	Izhikevich	0.95	0.01
Single spike	First spike	LIF	0.76	0.07
Single spike	Voting	LIF	0.72	0.07
Temporal	First spike	LIF	0.71	0.10
Temporal	Voting	LIF	0.75	0.06

Results are obtained from 31 independent runs with the best hyperparameter found for each configuration.

**Table 7:** Hyperparameter values found for the Iris Dataset.

**Config**.	**K**	**T**	**N**	***P*_*w*_**	** $P_{n}^{s}$ **	** $P_{c}^{s}$ **	** $P_{n}^{l}$ **	** $P_{c}^{l}$ **	***C*1**	***C*2**	***C*3**
Single - First - IZH	0.456	2.656	0.22	0.743	0.029	0.029	0.03	0.114	1.379	0.91	0.36
Single - Voting - IZH	0.456	2.656	0.22	0.743	0.029	0.029	0.03	0.114	1.379	0.91	0.36
Temporal - First - IZH	0.473	2.714	0.347	0.827	0.021	0.033	0.359	0.195	1.072	0.799	0.302
Temporal - Voting - IZH	0.517	3.753	0.169	0.84	0.032	0.012	0.196	0.068	0.629	0.694	0.335
Single - First - LIF	0.527	2.212	0.343	0.877	0.03	0.045	0.324	0.122	0.825	0.942	0.379
Single - Voting - LIF	0.402	2.465	0.327	0.879	0.03	0.026	0.196	0.097	0.504	0.719	0.344
Temporal - First - LIF	0.429	3.644	0.22	0.733	0.038	0.024	0.183	0.056	0.752	0.711	0.415
Temporal - Voting - LIF	0.515	3.144	0.2	0.804	0.026	0.037	0.33	0.187	0.701	1.383	0.35

**Figure 7:**
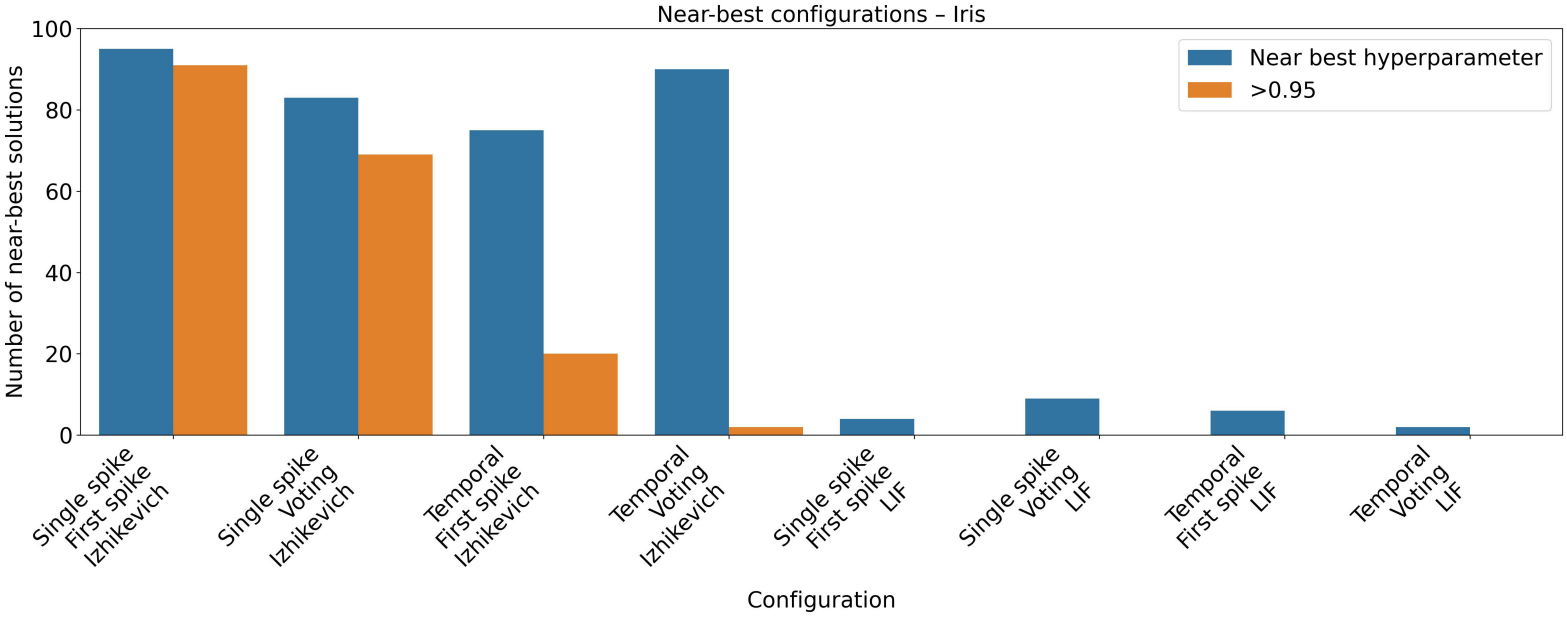
Near-best analysis for the Iris dataset. Hyperparameter optimization was performed using the Optuna framework, evaluating 100 different hyperparameter configurations for each combination of neuron model and encoding scheme. The plot shows the number of configurations that achieved a mean return within 95% of the best reward obtained during the optimization, as well as the number of configurations whose accuracy was higher than 0.95.

During the hyperparameter optimization, a clear difference emerged between the Izhikevich and LIF neuron models (see Figure 7). For the Izhikevich model, most hyperparameter configurations achieved very similar accuracy values, indicating a broad high-performing region in the hyperparameter space. In contrast, for the LIF model, only a small subset of configurations reached accuracy levels close to the best observed performance.

### 3.5 The Wine dataset

**Figure 8:**
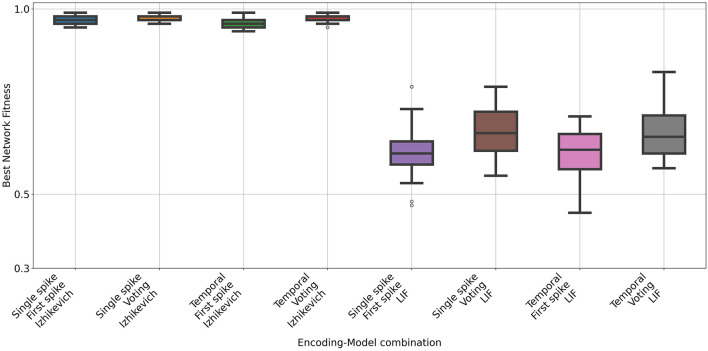
Accuracy of the spiking networks obtained for the Wine dataset. Each box shows the results of 31 independent runs with different input, out coding and neuron model. As in the previous task, configurations with the Izhikevich neuron model consistently performed better than those with LIF.

**Figure 9:**
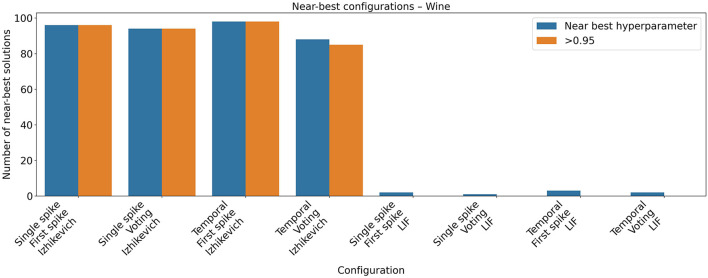
Near-best analysis for the Wine dataset. Hyperparameter optimization using Optuna evaluated 100 configurations for each neuron model and encoding scheme. The figure reports the number of configurations achieving performance within 95% of the best observed result, as well as those reaching an accuracy above 0.95.

The Wine dataset contains 178 instances of chemical analysis of different wines. For this dataset, the networks generated with the Izhikevich model obtained state-of-the art accuracy values with all encoding and decoding mechanisms (Figure 8, Tables 8, 9 for hyperparameter values). The LIF model, as in the experiments with the Iris dataset, produced worse results. As in the previous dataset a Mann-Whitney U test confirmed the difference. During the hyperparameter optimization, a pattern similar to that observed for the Iris dataset emerged for the Wine dataset (see Figure 9). Configurations using the Izhikevich neuron model exhibited a broad high-performing region in the hyperparameter space, whereas those using the LIF model showed a much narrower region of near-optimal solutions.

**Table 8:** Mean accuracy obtained in the Wine dataset for each configuration.

**Input coding**	**Output coding**	**Model**	**Mean**	**STD**
Single spike	First spike	Izhikevich	0.96	0.01
Single spike	Voting	Izhikevich	0.97	0.01
Temporal	First spike	Izhikevich	0.96	0.01
Temporal	Voting	Izhikevich	0.97	0.01
Single spike	First spike	LIF	0.61	0.07
Single spike	Voting	LIF	0.66	0.06
Temporal	First spike	LIF	0.61	0.06
Temporal	Voting	LIF	0.66	0.06

Results are obtained from 31 independent runs with the best hyperparameter found for each configuration.

**Table 9:** Hyperparameter values found for the Wine Dataset.

**Config**.	**K**	**T**	**I**	**N**	***P*_*w*_**	** $P_{n}^{s}$ **	** $P_{c}^{s}$ **	** $P_{n}^{l}$ **	** $P_{c}^{l}$ **	***C*1**	***C*2**	***C*3**
Single - First - IZH	0.552	2.976	0.195	0.873	0.034	0.028	0.331	0.195	0.67	1.364	0.377	
Single - Voting - IZH	0.412	3.039	0.244	0.797	0.022	0.042	0.182	0.151	1.289	1.113	0.364	
Temporal - First - IZH	0.504	2.814	0.268	0.872	0.027	0.033	0.157	0.087	1.348	1.131	0.339	
Temporal - Voting - IZH	0.421	3.32	0.265	0.842	0.035	0.041	0.094	0.081	1.111	0.516	0.354	
Single - First - LIF	0.524	2.52	0.266	0.794	0.038	0.014	0.293	0.149	1.182	0.862	0.408	
Single - Voting - LIF	0.405	3.425	0.192	0.851	0.034	0.034	0.118	0.184	1.356	1.299	0.387	
Temporal - First - LIF	0.408	2.859	0.284	0.897	0.035	0.05	0.053	0.18	0.833	1.476	0.368	
Temporal - Voting - LIF	0.443	3.371	0.327	0.888	0.039	0.015	0.039	0.144	1.342	1.019	0.379	

### 3.6 The Breast Cancer dataset

**Figure 10:**
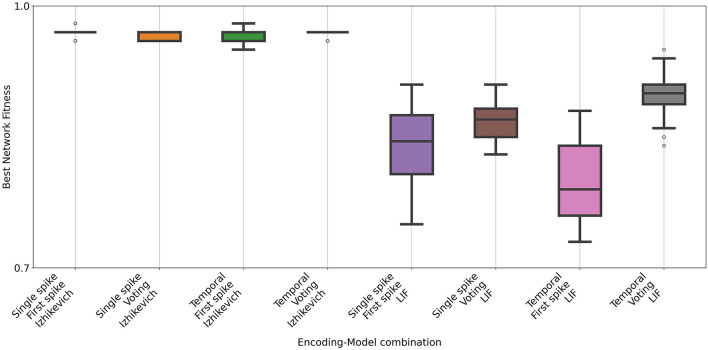
Classification accuracy of spiking neural networks on the Breast Cancer dataset. Each boxplot represents results from 31 independent runs using varying input encodings, output codings, and neuron models. Similar to the previous task, configurations employing the Izhikevich neuron model consistently outperformed those using the LIF model.

**Figure 11:**
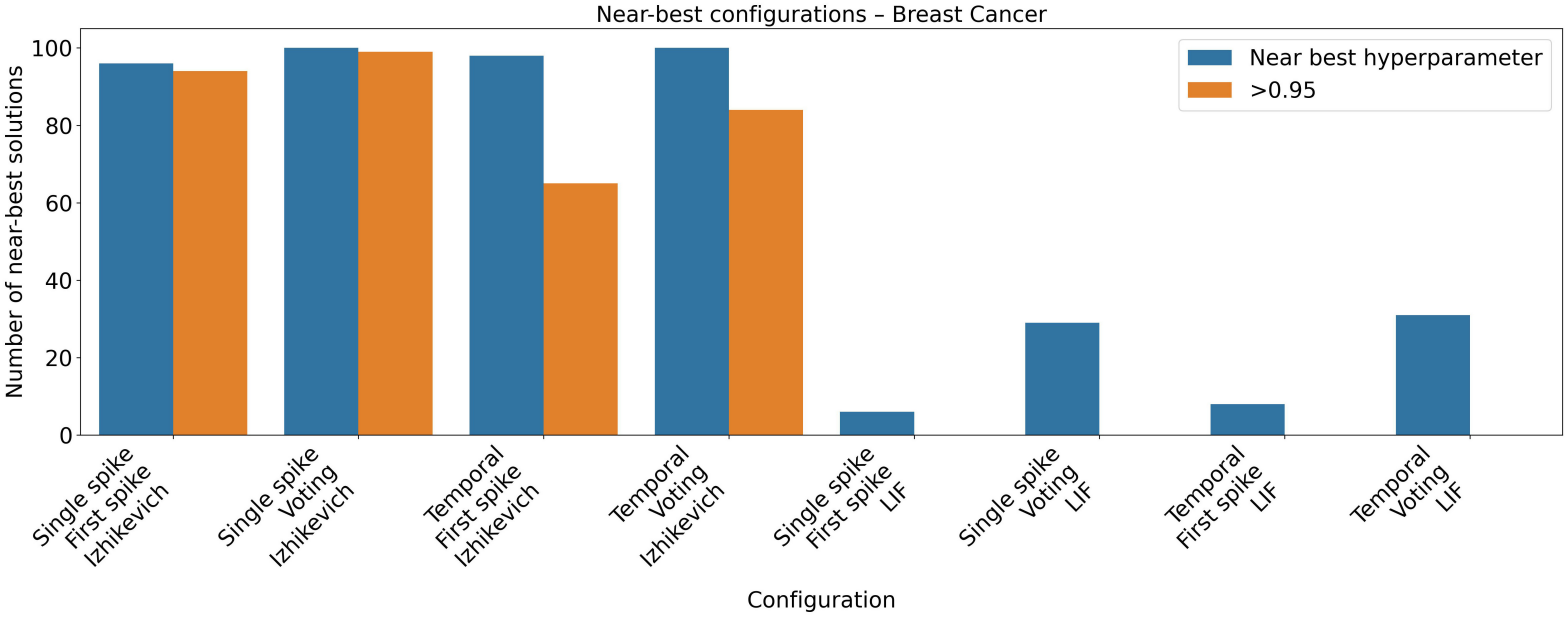
Near-best analysis for the Breast Cancer dataset. Results of the Optuna-based hyperparameter search (100 configurations per condition) illustrating the number of configurations within 95% of the best-performing solution and those achieving classification accuracy above 0.95, highlighting differences in robustness between neuron models.

The Breast Cancer has 269 instances of digitized image of a fine needle aspirate (FNA) of a breast mass. Again, experiments with the Izhikevich model produced state-of-the-art classifiers for the problem while experiments with the LIF model produced worse results (Figure 10, Tables 10, 11 for hyperparameters) as confirmed with a Mann-Whitney U test. During hyperparameter optimization for the Breast Cancer dataset, a similar trend was observed to that reported for the previous classification tasks (see Figure 11). Configurations employing the Izhikevich neuron model displayed a broad region of high-performing hyperparameters, whereas those using the LIF model were associated with a substantially narrower region of near-optimal solutions.

**Table 10:** Mean accuracy obtained in the Breast Cancer dataset for each configuration.

**Input coding**	**Output coding**	**Model**	**Mean**	**STD**
Single spike	First spike	Izhikevich	0.96	0.01
Single spike	Voting	Izhikevich	0.96	0.01
Temporal	First spike	Izhikevich	0.96	0.01
Temporal	Voting	Izhikevich	0.96	0.03
Single spike	First spike	LIF	0.84	0.04
Single spike	Voting	LIF	0.86	0.02
Temporal	First spike	LIF	0.79	0.04
Temporal	Voting	LIF	0.89	0.02

Results are obtained from 31 independent runs with the best hyperparameter found for each configuration.

**Table 11:** Hyperparameter values found for the Breast Cancer Dataset.

**Config**.	**K**	**T**	**N**	***P*_*w*_**	** $P_{n}^{s}$ **	** $P_{c}^{s}$ **	** $P_{n}^{l}$ **	** $P_{c}^{l}$ **	***C*1**	***C*2**	***C*3**
Single - First - IZH	0.453	3.584	0.344	0.774	0.035	0.047	0.343	0.137	1.382	1.219	0.471
Single - Voting - IZH	0.489	3.148	0.233	0.728	0.031	0.048	0.274	0.142	0.7	0.637	0.409
Temporal - First - IZH	0.424	3.907	0.242	0.723	0.03	0.016	0.293	0.106	0.873	0.982	0.382
Temporal - Voting - IZH	0.519	2.587	0.217	0.864	0.026	0.014	0.084	0.188	0.776	0.984	0.405
Single - First - LIF	0.498	2.067	0.211	0.88	0.034	0.032	0.246	0.13	1.218	1.139	0.382
Single - Voting - LIF	0.469	2.016	0.172	0.884	0.033	0.014	0.042	0.164	0.736	0.661	0.402
Temporal - First - LIF	0.445	2.263	0.201	0.76	0.027	0.023	0.13	0.096	0.727	0.697	0.356
Temporal - Voting - LIF	0.411	3.872	0.236	0.74	0.027	0.019	0.254	0.107	1.174	0.502	0.479

## 4 Discussion

We conducted a series of experiments using NEAT-generated spiking networks for both reinforcement learning and classification tasks, systematically varying neuron models and coding schemes. Specifically, we compared the widely used and computationally simple LIF model with the more biologically realistic Izhikevich model. Across all tasks, the Izhikevich model consistently outperformed the LIF model, with the exception of one task where both models achieved comparable results.

These findings underscore the importance of selecting an appropriate neuron model, an aspect that can be as critical as the choice of encoding scheme. Careful consideration of model configurations is essential, as performance can vary significantly depending on the specific task. This highlights the need for prototyping and systematic evaluation when designing neuromorphic algorithms. Simulation frameworks such as ANNarchy offer a valuable environment for exploring a wide range of configurations, enabling researchers to identify optimal setups tailored to individual problems.

The near-best analysis indicates that the reported results are not driven by a single narrowly tuned hyperparameter configuration. Instead, multiple high-performing solutions were found across the hyperparameter space, particularly for networks using the Izhikevich neuron model, which consistently exhibited broader regions of near-optimal performance. In contrast, configurations based on the LIF model often showed more restricted high-performing regions, suggesting greater sensitivity to precise hyperparameter selection. These findings strengthen confidence in the robustness of the observed performance differences and support the conclusion that they reflect systematic effects of neuron model dynamics rather than optimization artifacts.

Beyond the quantitative performance differences, the superior results obtained with the Izhikevich neuron model can be interpreted in light of its richer intrinsic dynamics. Unlike the LIF model, whose state is fully described by a single membrane potential variable, the Izhikevich model includes an additional recovery variable that introduces spike-frequency adaptation, bursting behavior, and history-dependent excitability. These mechanisms effectively endow individual neurons with a form of short-term memory and adaptive gain control, allowing their responses to depend not only on instantaneous input but also on recent spiking activity.

Such properties are particularly relevant in control and reinforcement learning tasks, where successful behavior often requires integrating information over time and responding robustly to delayed or noisy state transitions. In these tasks, the recovery variable may help stabilize control policies by preventing excessive firing and encoding temporal context through adaptive firing patterns. This interpretation is consistent with the observation that the performance gap between neuron models is most pronounced in tasks requiring sustained temporal coordination.

In classification tasks, the richer firing repertoire of the Izhikevich model may similarly enhance separability, enabling networks to exploit precise spike timing, transient bursts, or adaptive firing rates as discriminative features. Although the present study does not explicitly isolate the contribution of individual dynamical mechanisms, the consistent advantage of the Izhikevich model across diverse tasks suggests that neuronal adaptation play a functional role beyond mere biological realism.

Future work could investigate these hypotheses more directly by systematically disabling specific dynamical features of the Izhikevich model or by analyzing spiking statistics and adaptation profiles in trained networks. Such analyses would help clarify how intrinsic neuronal dynamics interact with evolutionary learning to shape effective computation in spiking neural networks.

A key strength of our study is its rigorous and systematic evaluation methodology. For every combination of neuron model, coding scheme, and task, we conducted an extensive hyperparameter search to ensure that performance comparisons were not biased by suboptimal configurations. Running the algorithm across 100 hyperparameter combinations for each pairing of neuron model and coding strategy was computationally intensive and required a substantial amount of time.

The superior performance of the Izhikevich model observed in our experiments supports the growing argument for advancing neuromorphic hardware beyond current limitations. Most existing platforms are optimized for simple neuron models such as LIF, prioritizing computational efficiency over biological realism. However, our findings demonstrate that more complex models, such as the Izhikevich neuron, can yield significantly better results across diverse tasks, suggesting that their use may be important for achieving higher levels of performance and adaptability in neuromorphic systems.

Under a standard forward Euler discretization, the computational cost of the Izhikevich neuron model is significantly higher than that of LIF model when measured in floating-point operations (FLOPs) per neuron per time step. A typical LIF update, consisting of a linear membrane potential equation with constant parameters, requires approximately seven floating-point operations, excluding threshold comparisons and reset assignments. In contrast, the Izhikevich model involves the numerical integration of two coupled differential equations, including a quadratic term in the membrane potential and an additional recovery variable. The corresponding Euler updates require approximately sixteen floating-point operations per time step, again excluding spike detection and reset logic. Consequently, the Izhikevich model incurs roughly a 2–2.5 × higher arithmetic cost per neuron per time step than the LIF model, reflecting its increased biophysical expressiveness at the expense of computational efficiency. However, recent approaches propose the use of posit arithmetic instead of floating-point representations to accelerate the computation of Izhikevich neural networks while preserving numerical precision (Fernandez-Hart et al., 2024).

The results presented in this study are specific to the evolutionary training method employed and may not necessarily generalize to other approaches, such as backpropagation with conjugate gradients. Furthermore, all comparisons were conducted using a fixed population size and a fixed number of generations to ensure a fair evaluation of the different configurations under identical computational constraints. It remains possible that the outcomes could vary under alternative computational budgets or training regimes. Also, we tested only a single parameter configuration for the Izhikevich model. Since the model's activation patterns are highly sensitive to parameter choices, different configurations may lead to significantly different results.

It is important to note that the temporal encoding strategy adopted in this study does not aim to faithfully reproduce biological latency coding. In many biological sensory systems, salient or high-confidence stimuli are associated with shorter response latencies, whereas longer delays often reflect increased uncertainty or the need for extended evidence accumulation. Such latency-based representations provide clear advantages in terms of reaction time and rapid decision making.

By contrast, our encoding maps feature magnitude directly to spike delay, resulting in higher values producing later spikes. This choice was primarily motivated by simplicity, rather than by biological plausibility. While this representation may sacrifice some of the reaction-time advantages observed in biological systems, it allows for a straightforward and interpretable temporal ordering of feature values that can be effectively exploited by evolutionary learning.

Importantly, the strong performance obtained with this encoding, particularly when combined with the Izhikevich neuron model, suggests that the networks are able to leverage temporal information even under non-biological latency mappings. Nevertheless, alternative encodings that invert this relationship, or that explicitly model uncertainty-dependent latency, represent a promising direction for future work and may further enhance performance, especially in time-critical tasks.

## Data Availability

The datasets presented in this study can be found in online repositories. The names of the repository/repositories and accession number(s) can be found below: https://github.com/bastianloyola/ANNarchy-NEAT.

## References

[B1] Abd Elaziz M. Dahou A. Abualigah L. Yu L. Alshinwan M. Khasawneh A. M. . (2021). Advanced metaheuristic optimization techniques in applications of deep neural networks: a review. Neural Comput. Applic. 33, 14079–14099. doi: 10.1007/s00521-021-05960-5

[B2] Akiba T. Sano S. Yanase T. Ohta T. Koyama M. (2019). “Optuna: A next-generation hyperparameter optimization framework,” in Proceedings of the 25th ACM SIGKDD International Conference on Knowledge Discovery and Data Mining, 2623–2631.

[B3] Auge D. Hille J. Mueller E. Knoll A. (2021). A survey of encoding techniques for signal processing in spiking neural networks. Neural Proc. Lett. 53, 4693–4710. doi: 10.1007/s11063-021-10562-2

[B4] Custode L. L. Mo H. Ferigo A. Iacca G. (2022a). Evolutionary optimization of spiking neural p systems for remaining useful life prediction. Algorithms 15:98. doi: 10.3390/a15030098

[B5] Custode L. L. Mo H. Iacca G. (2022b). “Neuroevolution of spiking neural p systems,” in International Conference on the Applications of Evolutionary Computation (Part of EvoStar) (Springer), 435–451. doi: 10.1007/978-3-031-02462-7_28

[B6] Elbrecht D. Schuman C. (2020). “Neuroevolution of spiking neural networks using compositional pattern producing networks,” in International Conference on Neuromorphic Systems 2020, 1–5. doi: 10.1145/3407197.3407198

[B7] Fernandez-Hart T. Knight J. C. Kalganova T. (2024). Posit and floating-point based Izhikevich neuron: a comparison of arithmetic. Neurocomputing 597:127903. doi: 10.1016/j.neucom.2024.127903

[B8] Frenkel C. Lefebvre M. Legat J.-D. Bol D. (2018). A 0.086-mm^2^ 12.7-pj/sop 64k-synapse 256-neuron online-learning digital spiking neuromorphic processor in 28-nm cmos. IEEE Trans. Biomed. Circuits Syst. 13, 145–158. doi: 10.1109/TBCAS.2018.2880425 30418919

[B9] Froese V. Hertrich C. (2023). “Training neural networks is np-hard in fixed dimension,” in Advances in Neural Information Processing Systems, 44039–44049.

[B10] Galván E. Mooney P. (2021). Neuroevolution in deep neural networks: current trends and future challenges. IEEE Trans. Artif. Intell. 2, 476–493. doi: 10.1109/TAI.2021.3067574

[B11] Haşegan D. Deible M. Earl C. D'Onofrio D. Hazan H. Anwar H. . (2022). Training spiking neuronal networks to perform motor control using reinforcement and evolutionary learning. Front. Comput. Neurosci. 16:1017284. doi: 10.3389/fncom.2022.1017284 36249482 PMC9563231

[B12] Izhikevich E. M. (2003). Simple model of spiking neurons. IEEE Trans. Neural. Netw. 14, 1569–1572. 18244602 10.1109/TNN.2003.820440

[B13] Jiang H. Lu J. Zhang C. Tang S. An J. Cheng L. . (2023). Multicore spiking neuromorphic chip in 180-nm with reram synapses and digital neurons. IEEE J. Emer. Selected Topics Circ. Syst. 13, 975–985. doi: 10.1109/JETCAS.2023.3325158

[B14] Kaveh M. Mesgari M. S. (2023). Application of meta-heuristic algorithms for training neural networks and deep learning architectures: a comprehensive review. Neural Proc. Lett. 55, 4519–4622. doi: 10.1007/s11063-022-11055-6 36339645 PMC9628382

[B15] Lee Y.-J. On M. B. Xiao X. Proietti R. Yoo S. B. (2022). Photonic spiking neural networks with event-driven femtojoule optoelectronic neurons based on izhikevich-inspired model. Opt. Express 30, 19360–19389. doi: 10.1364/OE.449528 36221716

[B16] Ma D. Jin X. Sun S. Li Y. Wu X. Hu Y. . (2024). Darwin3: a large-scale neuromorphic chip with a novel isa and on-chip learning. Natl. Sci. Rev. 11:nwae102. doi: 10.1093/nsr/nwae102 38689713 PMC11060491

[B17] Mishra V. Kane L. (2023). A survey of designing convolutional neural network using evolutionary algorithms. Artif. Intell. Rev. 56, 5095–5132. doi: 10.1007/s10462-022-10303-4

[B18] Neftci E. O. Mostafa H. Zenke F. (2019). Surrogate gradient learning in spiking neural networks: bringing the power of gradient-based optimization to spiking neural networks. IEEE Signal Process. Mag. 36, 51–63. doi: 10.1109/MSP.2019.2931595

[B19] Papavasileiou E. Cornelis J. Jansen B. (2021). A systematic literature review of the successors of “neuroevolution of augmenting topologies”. Evol. Comput. 29, 1–73. doi: 10.1162/evco_a_00282 33151100

[B20] Qiu H. Garratt M. Howard D. Anavatti S. (2018). “Evolving spiking neural networks for nonlinear control problems,” in 2018 IEEE Symposium Series on Computational Intelligence (SSCI) (IEEE), 1367–1373. doi: 10.1109/SSCI.2018.8628848

[B21] Schuman C. Rizzo C. McDonald-Carmack J. Skuda N. Plank J. (2022). “Evaluating encoding and decoding approaches for spiking neuromorphic systems,” in Proceedings of the International Conference on Neuromorphic Systems 2022, 1–9. doi: 10.1145/3546790.3546792

[B22] Schuman C. D. Kulkarni S. R. Parsa M. Mitchell J. P. Date P. Kay B. (2022). Opportunities for neuromorphic computing algorithms and applications. Nature Comput. Sci. 2, 10–19. doi: 10.1038/s43588-021-00184-y 38177712

[B23] Schuman C. D. Plank J. S. Bruer G. Anantharaj J. (2019). “Non-traditional input encoding schemes for spiking neuromorphic systems,” in 2019 International Joint Conference on Neural Networks (IJCNN) (IEEE), 1–10. doi: 10.1109/IJCNN.2019.8852139

[B24] Stanley K. O. Miikkulainen R. (2002). Evolving neural networks through augmenting topologies. Evol. Comput. 10, 99–127. doi: 10.1162/106365602320169811 12180173

[B25] Uludağ R. B. Çağdaş S. İşler Y. S. Şengör N. S. Aktürk İ. (2024). Bio-realistic neural network implementation on loihi 2 with izhikevich neurons. Neuromor. Comput. Eng. 4:024013. doi: 10.1088/2634-4386/ad5584

[B26] Vitay J. Dinkelbach H. Ü. Hamker F. H. (2015). ANNarchy: a code generation approach to neural simulations on parallel hardware. Front. Neuroinform. 9:19. doi: 10.3389/fninf.2015.00019 26283957 PMC4521356

